# Different Cytokine Patterns in *BMPR2*-Mutation-Positive Patients and Patients With Pulmonary Arterial Hypertension Without Mutations and Their Influence on Survival

**DOI:** 10.1016/j.chest.2022.01.019

**Published:** 2022-01-19

**Authors:** Max Schwiening, Emilia M. Swietlik, Divya Pandya, Keith Burling, Peter Barker, Oliver Y. Feng, Carmen M. Treacy, Susana Abreu, S. John Wort, Joanna Pepke-Zaba, Stefan Graf, Stefan J. Marciniak, Nicholas W. Morrell, Elaine Soon

**Affiliations:** a Cambridge Institute for Medical Research, University of Cambridge, Cambridge, England; b Department of Respiratory Medicine, Cambridge University Hospitals NHS Foundation Trust, Cambridge, England; c Royal Papworth Hospital NHS Foundation Trust, Papworth Road, Trumpington, Cambridge, England; d NIHR Cambridge BRC Core Biochemical Assay Laboratory (CBAL), Cambridge University Hospitals NHS Foundation Trust, Cambridge, England; e Statistical Laboratory, Centre for Mathematical Sciences, Cambridge, England; f National Heart and Lung Institute, Imperial College London, London, England; g Department of Hematology, University of Cambridge, Cambridge, England; h NIHR BioResource for Translational Research, Cambridge Biomedical Campus, Cambridge, England

To the Editor:

Pulmonary arterial hypertension (PAH) covers a range of life-limiting illnesses that are characterized by increased mean pulmonary artery pressures that, if untreated, lead to right heart failure and death. This is due to remodeling of the small-to-medium sized pulmonary vessels, which obstruct blood flow. PAH can be further categorized into idiopathic PAH (without any identifiable cause, 6th World Symposium class 1.1^1^) and heritable PAH (defined by mutations in specific genes, 6th World Symposium class 1.2^1^), the most common affecting bone morphogenetic protein receptor type II (*BMPR2*).^2^^,^^3^ It is known that patients who are BMPR2-mutation positive have worse cardiac indexes at presentation and a worse overall outcome compared with PAH without mutations.^4^ Possessing a BMPR2 mutation also creates a proinflammatory state, through loss of endothelial barrier function^5^ and loss of antioxidant capability.^6^ This then begs the question as to whether the mutation-positive groups have different underlying pathogenetic mechanisms and require different biomarkers and treatments, analogous to how patients with epidermal growth factor receptor-mutation-positive non-small cell lung cancer respond to tyrosine kinase inhibition, although the majority of patients with non-small cell lung cancer do not.

We wondered if the broad proinflammatory phenotype seen in idiopathic PAH^7^ would be different in the heritable PAH subgroup. To answer questions like these, the National Cohort Study of Idiopathic and Heritable PAH (http://www.ipahcohort.com/) was set up in 2014.

## Methods

All patients with a confirmed diagnosis of idiopathic or heritable PAH were recruited prospectively from 2014 onwards. 56 healthy control subjects were also recruited from among sex- and age-matched staff, who had no personal history or family history of pulmonary hypertension. Idiopathic PAH was defined as a mean pulmonary artery pressure of ≥ 25 mm Hg at rest with a pulmonary capillary wedge pressure of ≤ 15 mm Hg with no underlying cause for PAH.^8^ Heritable PAH was defined as familial PAH or where a pathogenic mutation is present.^9^ We note these classifications differ slightly from the most recent guidelines, because the cohort was established in 2014, predating the 6th World Symposium. All patients systematically underwent next-generation paired-end whole-genome sequencing with the use of the Illumina HiSeq2500.^10^ All patients who were carrying the BMPR2-mutation were identified from the patients with heritable PAH and screened to determine if they had contributed plasma samples. For every patient who tested BMPR2-mutation positive, a patient with PAH without any mutations who was the next admission in the database was selected as a disease (positive) control. Patients without mutations were defined as patients with PAH with no family history in whom no mutation has been found in either established (*BMPR2, EIF2AK4, SMAD1/4/8, CAV1, KCNK, ENG, ALK1, TBX4*) or new PAH risk genes (*GDF2, AQP1, ATP13A3, SOX17, ABCC8, KLK1, GCCX, KDR, BMP10*). This led to the recruitment of 54 patients who carried the BMPR2-mutation and 54 patients with PAH who did not have any mutations. We systematically collected demographic data, diagnostic hemodynamic measurements, survival status at December 31, 2018, and N-terminal pro-B type natriuretic peptide (NT-proBNP) levels. The outcome of all patients was known. The study was approved by the National Research Ethics Service (REF:13/EE/0325, 13/EE/0203). All participants gave informed written consent. The following analytes (IL-2, IL-6, IL-8, IL-10, tumor necrosis factor-alpha [TNF-α], interferon-gamma, vascular endothelial growth factor-A

[VEGF-A], granulocyte colony-stimulating factor [G-CSF]) were measured with the use of a custom multiplex assay (Meso Scale Discovery; Gaithersburg, MD) and performed to manufacturer’s protocols (https://www.mesoscale.com/en/products/u-plex-immuno-oncology-group-1-human-111-plex-k15342k/). These analytes were chosen for the following reasons: IL-2, IL-6, IL-8, and IL-10 have previously shown to influence survival in PAH.^7^ TNF-α is proven to interact with BMPR2 signalling.^5^ The VEGF pathway has been implicated^11^ and G-CSF has previously been shown potentially to be protective^12^ in rodent models of PAH. Data were checked for adherence to a parametric distribution with the Kolmogorov-Smirnoff method and compared with *t*-tests (for parametric data) and Mann-Whitney tests (for nonparametric data). The Benjamini-Hochberg test was also applied to the comparisons of analytes in the multiplex assay to control the false discovery rate. The relationship between cytokines and survival subsequently was interrogated with the use of Kaplan-Meier analyses, receiver operating characteristic curves, and Cox proportional hazard models.

## Results

Patients who carried the *BMPR2-*mutation were younger and had worse hemodynamic indexes at presentation. Table 1 provides a summary of the demographics and cytokine levels in all patient groups and control subjects. Both patients who carried the *BMPR2-*mutation and patients without mutations had high levels of IL-6, IL-8 and TNF-α compared with control subjects. For example, the median level for IL-6 was 0.70 pg/mL (interquartile range, 0.60 to 0.90 pg/mL) in control subjects compared with 1.60 pg/mL (interquartile range, 1.10 to 2.10 pg/mL) for patients who were *BMPR2*-mutation positive and 1.35 pg/mL (interquartile range, 0.90 to 2.23 pg/mL) in patients with PAH without mutations (*P* < .0001 for both). Interestingly, patients without mutations had much higher VEGF-A levels (48.5 pg/mL [interquartile range, 33.3 to 93.8 pg/mL]) compared with patients who carried the *BMPR2-*mutation (35.0 pg/mL [interquartile range, 26.0 to 54.0 pg/mL]; *P* = .0008).

**Table 1:** Baseline Characteristics and Biomarker Levels of All Study Participants

Parameter	Control Subjects (Healthy Volunteers)	Pulmonary Arterial Hypertension		
		Total	Bone Morphogenetic Protein Receptor Type-2 Mutation	Pulmonary Arterial Hypertension Sans Mutation
No.	56	108	54	54
Age, y	36.63 (12.50)	43.72 (12.76)	41.25 (13.38)^a^	46.10 (11.75)
Male:female	1:2.11	1:2.50	1:1.95	1:3.31
Mean pulmonary artery pressures, mm Hg	N/A	56.67 (11.20)	60.76 (12.33)^b^	52.73 (8.35)
Cardiac index, L/min/m^2^	N/A	2.14 (0.67)	1.99 (0.60)^a^	2.27 (0.70)
IL-6, pg/mL	0.70 (0.60-0.90)	1.40 (1.00-2.10)^c^^d^	1.60 (1.10-2.10)^c^^d^	1.35 (0.90-2.23)^c^^d^
IL-8, pg/mL	2.90 (2.50-3.60)	3.70 (2.70-6.20)^e^^d^	3.60 (2.90-5.50)^e^^d^	3.85 (2.53-6.58)^d^^f^
IL-10, pg/mL	0.30 (0.30-0.40)	0.40 (0.30-0.50)^d^^f^	0.35 (0.30-0.50)	0.40 (0.30-0.50)^d^^f^
Tumor necrosis factor-α, pg/mL	1.90 (0.50-2.28)	2.60 (2.10-3.20)^c^^d^	2.50 (2.10-2.90)^c^^d^	3.00 (2.00-3.80)^c^^d^
Vascular endothelial growth factor-A, pg/mL	30.0 (25.0-38.0)	40.0 (29.3-71.8)^c^^d^	35.0 (26.0-54.0)^a^^d^	48.5 (33.3-93.8)^c^^d^
G-CSF, pg/mL	12.0 (10.1-15.1)	13.1 (11.3-17.0)	13.4 (11.5-17.4)^d^^f^	12.7 (11.1-16.6)
Interferon-γ, pg/mL	10.3 (7.5-13.2)	11.7 (8.9-16.5)^d^^f^	11.5 (8.6-16.4)	12.2 (8.9-17.0)^d^^f^
N-terminal pro-B type natriuretic peptide, pg/mL	N/A	234 (84-904)	200 (73-790)	274 (88-956)

Data are given as mean (standard deviation) or median (interquartile range), unless otherwise indicated. IL-2 levels were also assayed but were below/at the limit of detection (1.50 pg/mL) for 150 of 164 samples. G-CSF = granulocyte colony stimulating factor; N/A = not available.

a *P* < .05 compared with pulmonary arterial hypertension patients without mutations with the use of t-tests (for parametric data) and Mann-Whitney tests (for nonparametric data).

b *P* < .001 compared with pulmonary arterial hypertension without mutations with the use of *t*-tests (for parametric data) and Mann-Whitney tests (for nonparametric data).

c *P* < .001 compared with control subjects with the use of *t*-tests (for parametric data) and Mann-Whitney tests (for nonparametric data).

d *P* reaches significance as determined by Benjamini-Hochberg testing with a false discovery rate of 0.1.

e *P* < .01 compared with control subjects with the use of t-tests (for parametric data) and Mann-Whitney tests (for nonparametric data).

f *P* < .05 compared with control subjects with the use of t-tests (for parametric data) and Mann-Whitney tests (for nonparametric data).

**Figure 1:**
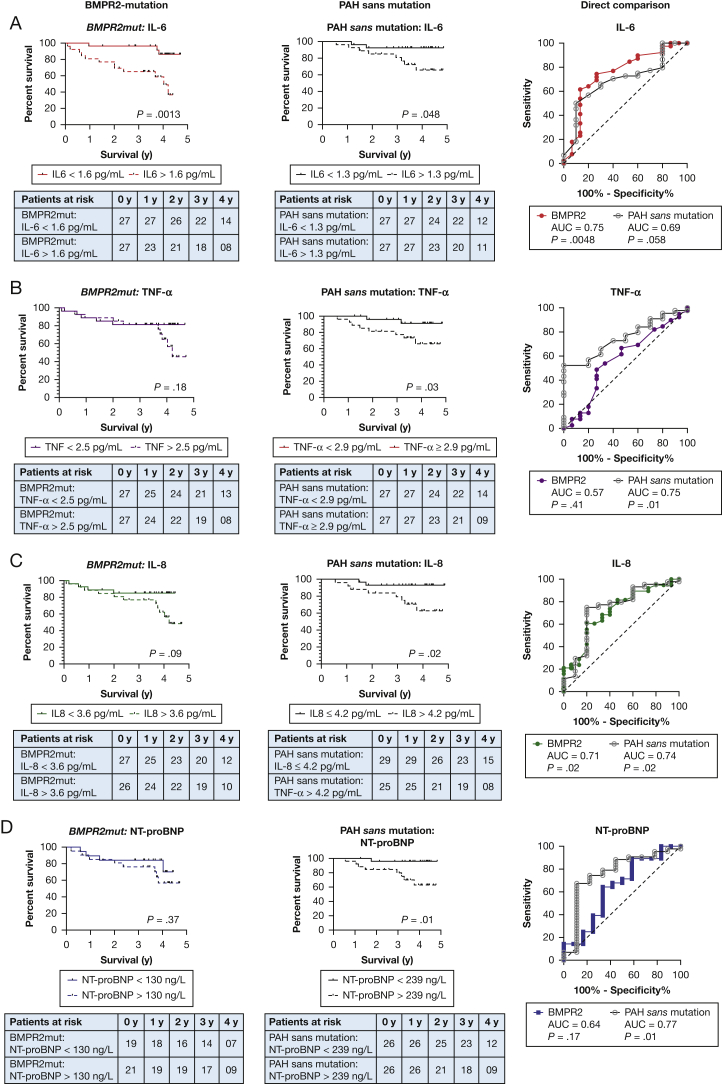
A-D, Graphs show survival curves based on Kaplan-Meier analyses and receiver operating curves. Rows show analyses based on IL-6 (A), TNF-α (B), IL-8 (C), and NT-proBNP (D). The leftmost column shows analyses in BMPR2-mutation-positive patients; the middle column shows analyses in patients with PAH without mutations, and the rightmost column shows the ROC curve that enables a direct comparison in both groups of patients. For A-B, log-rank *P* values are shown. For C, the *P* values shown relate to the significance of the AUC compared with the “line of no discrimination.” For ease of reading, the BMPR-2 mutation data are presented in color, and the PAH without mutation data are presented in black. AUC = area under the curve; BMPR2 = bone morphogenetic protein receptor type-2; NT-proBNP = N-terminal pro-B type natriuretic peptide; PAH = pulmonary arterial hypertension; ROC = receiver operating characteristic; TNF = tumor necrosis factor;

For patients who carried the *BMPR2*-mutation, IL-6 was a truly significant predictor of death; the 3-year cumulative survival for patients with IL-6 of ≥ 1.6 pg/mL was 65% compared with 96% for patients with an IL-6 of < 1.6 pg/mL (*P* = .0013; Fig 1A). For patients with PAH without mutations, IL-6 also proved to be a significant discriminator for survival, but to a lesser degree. The cumulative survival for patients with an IL-6 ≥ 1.3 pg/mL at 3 years was 81% compared with 92% for patients with an IL-6 < 1.3 pg/mL (*P* = .048). The reverse pattern applies to TNF-α and IL-8. In patients without mutations, TNF-α and IL-8 were capable of discriminating for survival (Fig 1B and C), but these effects were not seen in patients who carried the *BMPR2*-mutation.

Interestingly, the most commonly used serum biomarker, NT-proBNP, does not predict death in our cohort of *BMPR2*-mutation carrying patients (Fig 1D). To ensure that this was not an error, the survival analyses were redone with the use of tertiles of NT-proBNP levels and also with European Respiratory Society-validated cutoff values.^13^ No matter what cutoff value was used, NT-proBNP levels failed to discriminate for survival in patients who carried the *BMPR2*-mutation. This is supported by the receiver operating characteristic curves (Fig 1); the area under curve for IL-6 in patients who are *BMPR2*-mutation positive is 0.75 (*P* = .0048) vs 0.64 for NT-proBNP (*P* = .17). Conversely the area under the curve for NT-proBNP in patients with PAH without mutations is 0.77 (*P* = .01) vs 0.69 for IL-6 (*P* = .058).

Although simple and intuitive, Kaplan-Meier analyses dichotomizes the data and is unable to account for confounders. Therefore, to complement this we have also constructed three Cox proportional hazards models:

1. Model 1 considers the variables mutation status, IL-6, NT-proBNP, prevalent status, TNF-α, and IL-8, with the outcome being survival. IL-6, NT-proBNP, TNF-α, and IL-8 are considered as continuous variables while mutation status and prevalent status are binary.
2. Model 2 considers all the variables in Model 1 with an interaction term between mutation status and IL-6 levels (annotated as mutation:IL6).
3. Model 3 considers all the variables in Models 1 and 2 with an interaction term between mutation status and NT-proBNP levels (annotated as mutation:NT-proBNP).

The hazard ratios for the models are summarized in Table 2. In all three models, the BMPR-2 positivity, increased IL-6, and increased NT-proBNP levels were associated with an increased risk of death. It is likely, but not certain, that there is an interaction between BMPR2-mutation positivity and IL-6 (analysis of variance comparison: *P =* .08). It is very likely that there is no interaction between BMPR-2 positivity and NT-proBNP (analysis of variance comparison: *P =* .926). This is also supported by the Akaike’s information criterion, which is lowest in model 2 (lower Akaike’s information criterion corresponding to a better fit).

**Table 2:** Cox Regression Models of Variables That Influence Survival

Variable	Hazard Ratio		
	Model 1	Model 2	Model 3
Presence of BMPR2-mutation	3.38^a^	1.153	3.529^b^
IL-6	1.318^a^	1.172	1.317^a^
NT-proBNP	1.0005^a^	1^a^	1^b^
Prevalent status	0.9267	1.226	0.929
Tumor necrosis factor-α	1.126	1.099	1.126
IL-8	1.051	1.065^b^	1.051
Mutation:IL-6	N/A	1.71^b^	N/A
		(2.94)^b^	
Mutation:NT-proBNP	N/A	N/A	1
			(1)
Akaike information criterion	173	171	174
Analysis of variance comparison	Basis for comparison	0.08	0.926

For models 2 and 3, the estimated hazard ratios in rows 2 to 3 are those associated with per unit increase in IL-6 and NT-proBNP, respectively, for patients who are mutation-negative. By multiplying these values by the hazard ratios for the corresponding interaction terms, we can obtain estimates of the hazard ratios that are associated with IL-6 and NT-proBNP for patients who are BMPR2 mutation-positive (these data are provided in parentheses in the row below). AIC = Akaike information criterion; BMPR2 = bone morphogenetic protein receptor type-2; N/A = not available; NT-proBNP = N-terminal pro-B type natriuretic peptide.

a *P* < .05

b *P* < .1

The cohort was a mixed one, but of predominantly prevalent patients (76.9% or 83 of 108 patients). There were 12 incident patients in the BMPR2-mutation-positive cohort (total of 54) and 13 incident patients in the PAH sans mutation cohort (total of 54), so there was no difference in the proportion of incident patients between cohorts. In the Cox analyses, prevalent status appears not to have a significant impact on survival; however, this was likely due to the small numbers.

## Discussion

A potential difficulty in the use of IL-6 as a prognostic marker would be deciding what cutoff level to choose. Levels of IL-6 in PAH have been described previously and vary from 66 pg/mL (1995^14^) to 20 pg/mL (2010^7^) to 1.4 to 1.62pg/mL (2020^15^). This may mean that a standard assay needs to be adopted universally for measurement of IL-6 to make a standard cutoff valid and useful. The inclusion of different categories of type I PAH (notably connective tissue disease associated PAH) also increases the median levels for IL-6. Simpson et al^15^ reported median levels of 2.24 pg/mL (interquartile range, 1.09 to 4.33 pg/mL) in connective tissue disease associated PAH and 1.62 pg/mL (interquartile range, 0.72 to 2.94 pg/mL) in idiopathic PAH, which implies that IL-6 levels are disease- and genotype-specific. We suggest a cutoff of 1.6 pg/mL as being useful prognostically in both *BMPR2*-positive and PAH without mutations (3-year survival in the whole cohort of 94.9% vs 70.1%; *P* = .0002). Clearly, this requires validation in an independent patient group.

This study raises the possibility of different “driving” inflammatory components in *BMPR2*-mutation-positive PAH vs PAH without mutations. IL-6 appears to be implicated more heavily in BMPR2-associated PAH while TNF-α and IL-8 are more prominent in PAH sans mutations. TNF-α can suppress BMPR-II signaling^5^ directly, and it may be that the *BMPR2-*mutation-positive cohort does not require this because they already have constitutive genetic loss of BMPR-II.

This is the first study to show that prognostic biomarkers and, by inference, treatments could be genotype-specific in PAH. We propose an IL-6 level of 1.6 pg/mL as being useful in both BMPR2-associated and PAH without mutations. IL-6 is the most discriminating biomarker for death in *BMPR2*-mutation-positive PAH, and NT-proBNP fails in this regard. NT-proBNP outperforms IL-6 in PAH without mutations. We postulate that BMPR2-mutation-positive PAH may have a different driving inflammatory component and respond to different treatments compared with patients with PAH without mutations.
